# ﻿Comparison of seven complete mitochondrial genomes from *Lamprologus* and *Neolamprologus* (Chordata, Teleostei, Perciformes) and the phylogenetic implications for Cichlidae

**DOI:** 10.3897/zookeys.1184.107091

**Published:** 2023-11-15

**Authors:** Jiachen Wang, Jingzhe Tai, Wenwen Zhang, Ke He, Hong Lan, Hongyi Liu

**Affiliations:** 1 Co-Innovation Center for Sustainable Forestry in Southern China, College of Life Sciences, Nanjing Forestry University, Nanjing 210037, China Nanjing Forestry University Nanjing China; 2 Institute of Environmental Sciences, Ministry of Ecology and Environment of China State Environmental Protection Scientific Observation and Research Station for Ecological Environment of Wuyi Mountains Research Center for Biodiversity Conservation and Biosafety, Nanjing 210042, China Institute of Environmental Sciences, Ministry of Ecology and Environment of China State Environmental Protection Scientific Observation and Research Station for Ecological Environment of Wuyi Mountains Research Center for Biodiversity Conservation and Biosafety Nanjing China; 3 Zhejiang Agriculture and Forestry University, Hangzhou 311300, China Zhejiang Agriculture and Forestry University Hangzhou China; 4 Zhejiang Open University, Hangzhou 310012, China Zhejiang Open University Hangzhou China

**Keywords:** Cichlidae, *Lamprologus*, mitogenome, *Neolamprologus*, phylogenetic analyses

## Abstract

In this study, mitochondrial genomes (mitogenomes) of seven cichlid species (*Lamprologuskungweensis*, *L.meleagris*, *L.ornatipinnis*, *Neolamprologusbrevis*, *N.caudopunctatus*, *N.leleupi*, and *N.similis*) are characterized for the first time. The newly sequenced mitogenomes contained 37 typical genes [13 protein-coding genes (PCGs), two ribosomal RNA genes (rRNAs) and 22 transfer RNA genes (tRNAs)]. The mitogenomes were 16,562 ~ 16,587 bp in length with an A + T composition of 52.1~58.8%. The cichlid mitogenomes had a comparable nucleotide composition, A + T content was higher than the G + C content. The AT-skews of most mitogenomes were inconspicuously positive and the GC-skews were negative, indicating higher occurrences of C than G. Most PCGs started with the conventional start codon, ATN. There was no essential difference in the codon usage patterns of these seven species. Using Ka/Ks, we found the fastest-evolving gene were *atp8*. But the results of p-distance indicated that the fastest-evolving gene was *nad6.* Phylogenetic analysis revealed that *L.meleagris* did not cluster with *Lamprologus* species, but with species from the genus *Neolamprologus*. The novel information obtained about these mitogenomes will contribute to elucidating the complex relationships among cichlid species.

## ﻿Introduction

Cichlids (Teleostei: Perciformes: Cichlidae) are widely distributed across the Neotropics, Africa, the Middle East, Madagascar, as well as southern India and Sri Lanka (Smith et al. 2008; López-Fernández et al. 2010). They stand out as one of the most species-diverse groups of acanthomorphs. Kullander (1998) divided the family Cichlidae into eight subfamilies: Astronotinae, Cichlasomatinae, Cichlinae, Etroplinae, Geophaginae, Heterochromidinae, Pseudocrenilabrinae, and Retroculinae. The ninth subfamily, the Ptychochrominae, was later recognized by Sparks and Smith (2004). Cichlids gained recognition as a prominent model species for the study of evolutionary biology due to the numerous species, diverse genetics, distinct evolutionary lineages, and significant ecological and morphological divergences (Kocher 2004; Schwarzer et al. 2015; Reis et al. 2016; Nam et al. 2021).

African cichlids (subfamily Pseudocrenilabrinae) boasted an abundant variety of more than 2000 species (Brawand et al. 2014; Astudillo-Clavijo et al. 2022). Biologists have long been fascinated by the diversity of cichlids in the East African cichlid radiation (EAR), which has promoted high levels of endemism in the Lakes Tanganyika, Malawi, and Victoria (Kornfield and Smith 2000). Lake Tanganyika is a deep tropical and large Rift Valley lake with an age of 9–12 million years (Irisarri et al. 2018). It has the most diverse species of cichlid fish in terms of morphology, ecology, and behavior, including several mouth-brooding and substrate-spawning lineages (Takahashi 2003; Salzburger 2009). The cichlid fauna of Lake Tanganyika is dominated by lamprologine cichlids, which colonized most lacustrine habitats, but most often inhabits the littoral zone (Sturmbauer et al. 2010). Although classified as a single tribe, lamprologine cichlids exhibit significant diversity in morphology, ecology, and behavior. *Lamprologuskungweensis*, *Lamprologusmeleagris*, *Lamprologusornatipinnis*, *Neolamprologusbrevis*, *Neolamprologuscaudopunctatus*, *Neolamprologusleleupi*, and *Neolamprologussimilis* are among the smallest species within the lamprologine cichlids, small enough to live inside the empty shells of gastropod mollusks (Sturmbauer et al. 2010). These species are regarded as a highly valuable ornamental species in the aquatic trade industry due to their ease of maintenance and handling in aquariums (Nam et al. 2021).

The genera *Lamprologus* and *Neolamprologus* can be difficult to distinguish due to their similar morphology, ecology, and behavior. As discussed by Stiassny (1991), meristic and morphometric measurements, osteology, and dentition were insufficient to differentiate between the species, as many of these traits were homoplastic. Furthermore, there might be instances of ancient ancestral polymorphism, introgressive hybridization, or lack of diagnostic synapomorphic characters among certain species within these two genera, further complicating their classification (Sturmbauer et al. 2010; Gante et al. 2016). Therefore, additional method, like molecular analysis might be required for more accurate classification.

Mitochondria are organelles found in most eukaryotic cells that play a critical role in energy production (Hebert et al. 2010). The mitochondrial genome (mitogenome) of acanthomorph fishes is usually a circular, double-stranded molecule that ranges from 16 to 23 kbp in size. It typically contains 13 protein-coding genes (PCGs), two ribosomal RNA genes (rRNAs), 22 transfer RNA genes (tRNAs), and one control region (CR) (Iwasaki et al. 2013). Mitogenomes have the characteristics of high evolutionary rate, matrilineal inheritance, low molecular weight, simple structure, and ease of amplification, which makes them a reliable marker for studying phylogenetics (Ye et al. 2022; Wang et al. 2023). Mitogenome components, such as *nad2* or *rrnL*, are widely used for phylogenetic analyses (Sturmbauer et al. 2010; Schwarzer et al. 2015). Although partial mitochondrial sequences can offer some insights into evolutionary relationships, they are limited in their ability to provide a comprehensive understanding due to the absence of information such as gene rearrangement, genetic code changes, replication, and transcriptional regulation patterns. Therefore, complete mitogenome sequences can be more beneficial as they can provide improved resolution and sensitivity for investigating evolutionary relationships (Li et al. 2019; Fiteha et al. 2023; Wang et al. 2023).

In this study, we report the complete mitogenome organizations and characteristics of seven species (*L.kungweensis*, *L.meleagris*, *L.ornatipinnis*, *N.brevis*, *N.caudopunctatus*, *N.leleupi*, and *N.similis*). We also performed a phylogenetic analysis of the seven complete mitogenomes obtained in this study with the published complete cichlid mitogenomes. We hope that our study can enable better comprehension of cichlid biodiversity and expand genetic resources for future cichlid comparisons.

## ﻿Materials and methods

### ﻿Sample collection and DNA extraction

The seven species are commonly sold as ornamental fish and can be found in many pet markets. Specimens were obtained from the Qiqiaoweng pet market in Nanjing, Jiangsu province, China. The specimens were identified using morphological characteristics described in FishBase (https://www.fishbase.de/). No fish were sacrificed during this study. The fish were reared at the Laboratory of Animal Molecular Evolution, Nanjing Forestry University. Total genomic DNA was extracted from each fin using a FastPure Cell/Tissue DNA Isolation Mini Kit (Vazyme, Nanjing, China), and stored at –80 °C for future use.

### ﻿Genome sequencing, assembly, and annotation

Seven complete mitogenomes were sequenced on an Illumina platform (Personalbio Nanjin, China) using total genomic DNA. The genomic DNA was used to generate an Illumina library with an insert size of 400 bp. The clean data were then assembled in Geneious Prime 2022 software, using *Lamprologussignatus* (MZ427900.1) as a template. The mitogenomes were assembled and manually revised using DNAstar v. 7.1 (Madison, WI, USA).

Conservative domains were detected using BLAST (https://www.ncbi.nlm.nih.gov/Structure/cdd/wrpsb.cgi) and MITOS WebServer (http://mitos.bioinf.uni-leipzig.de/index.py) (Bernt et al. 2013). Maps of the mitogenomes were constructed using CGView (https://cgview.ca/) (Stothard and Wishart 2005). MEGA X was used for base composition analysis, relative synonymous codon usage (RSCU) analysis, pairwise relative genetic distance (p-distance) calculation, as well as non-synonymous (Ka) and synonymous substitutions (Ks) analysis (Fay and Wu 2003; Kumar et al. 2016). Composition skew values were calculated using the following formulas: “AT-skew = (A − T) / (A + T) GC-skew = (G − C) / (G + C)” (Perna and Kocher 1995).

### ﻿Phylogenetic analysis

Phylogenetic analysis was conducted using the sequences of 13 PCGs and two rRNA genes from the complete mitogenomes of 105 species, including seven species from this study (Suppl. material 1). *Channaandrao* and *Hyphessobryconsweglesi* were selected as outgroups, while the remaining specimens belonged to the Cichlidae family. Phylogenetic analysis was conducted using maximum likelihood (ML) and Bayesian inference (BI) methods with PhyloSuite v. 1.2.3 software package (Zhang et al. 2020; Xiang et al. 2023). All genes were aligned using MAFFT v. 7.313, and the best-fit substitution model and partitioning scheme were determined using ModelFinder. ML phylogenies were inferred using IQ-TREE with the Edge-linked partition model for 5000 ultrafast bootstraps (Minh et al. 2013; Nguyen et al. 2015). BI phylogenies were inferred using MrBayes v. 3.2.7a with a partition model (Ronquist et al. 2012). The analysis consisted of two parallel runs with 2,000,000 generations each, and the initial 25% of sampled data was discarded as burn-in. The trees were visualized and edited using iTOL v. 6 (Letunic and Bork 2021).

## ﻿Results and discussion

### ﻿Genome organization and composition

**Figure 1.**
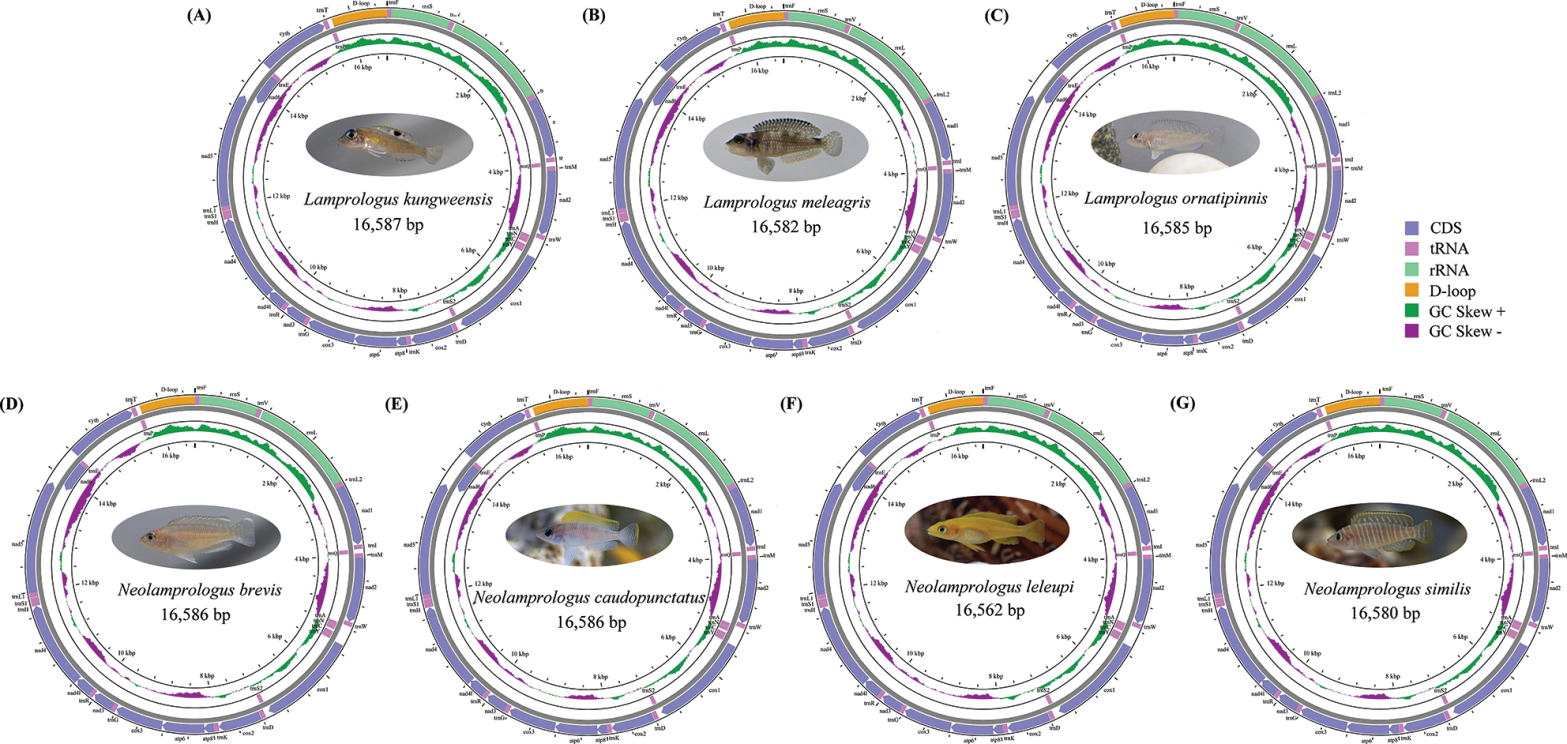
The gene maps of the seven newly sequenced mitogenomes. Different gene types are shown in different colors.

Seven complete mitogenomes covering two genera were obtained. *L.kungweensis* (16,587 bp), *L.meleagris* (16,582 bp), *L.ornatipinnis* (16,585 bp), *N.brevis* (16,586 bp), *N.caudopunctatus* (16,586 bp), and *N.similis* (16,580 bp) had similar lengths, while *N.leleupi* had the shortest length at 16,562 bp (Fig. 1) (accession numbers: OP805601.1, OP805600.1, OQ076695.1, OP930818.1, OP930816.1, OP930817.1, and OP930815.1). The seven mitogenomes possessed the typical gene composition found in most bony fish, including 13 PCGs, 22 tRNAs, two rRNAs, and a CR. Among these genes, 12 PCGs, 14 tRNA genes, and two rRNA genes, were located on the major strand (H-strand), while the remaining eight tRNA genes and a PCG were encoded on the minor strand (L-strand). The gene order of these mitogenomes was identical to that of previously published species *L.signatus* (MZ427900.1) and *Neolamprologusbrichardi* (AP006014.1) (Nam et al. 2021). Seventeen intergenic regions of the same length were observed between the mitochondrial regions of species *L.kungweensis*, *L.meleagris*, *L.ornatipinnis*, *N.brevis*, with lengths ranging from 10 bp (between *atp8* and *atp6*) to 35 bp (between *trnN* and *trnC*). However, *N.caudopunctatus* exhibited a 24 bp intergenic region between *trnV* and *rrnL*, and *N.similis* displayed a 38 bp intergenic region at the same location. The *trnC* and *trnY* of *N.similis* overlapped by 1 bp, whereas there was no overlap in this region in the other six species. In addition, *N.leleupi* had one more intergenic region (24 bp between *cox1* and *trnS2*) than other species (Table 1).

**Table 1.** Features of the mitogenomes of *L.kungweensis*, *L.meleagris*, *L.ornatipinnis*, *N.brevis*, *N.caudopunctatus*, *N.leleupi*, and *N.similis*.

Gene	Position		Size (bp)	Intergenic Nucleotides	Codon		Strand
	From	To			Start	Stop	
*trnF*	1/1/1/1/1/1/1	69/69/69/69/69/69/69	69/69/69/69/69/69/69	0/0/0/0/0/0/0			H
*rrnS*	70/70/70/70/70/70/70	1013/1012/1013/1012/1012/1013/1015	944/943/944/943/943/944/946	0/0/0/0/0/0/0			H
*trnV*	1014/1013/1014/1013/1013/1014/1016	1085/1084/1085/1084/1084/1085/1087	72/72/72/72/72/72/72	0/0/0/0/0/0/0			H
*rrnL*	1108/1107/1108/1107/1109/1108/1126	2776/2776/2776/2777/2778/2775/2777	1669/1670/1669/1671/1670/1668/1652	22/22/22/22/24/22/38			H
*trnL2*	2777/2777/2777/2778/2779/2776/2778	2850/2850/2850/2851/2852/2849/2851	74/74/74/74/74/74/74	0/0/0/0/0/0/0			H
*nad1*	2851/2851/2851/2852/2853/2850/2852	3825/3825/3825/3826/3827/3824/3826	975/975/975/975/975/975/975	0/0/0/0/0/0/0	ATG/ATG/ATG/ATG/ATG/ATG/ATG	TAG/TAA/TAG/TAG/TAG/TAG/TAG	H
*trnI*	3829/3829/3829/3830/3831/3828/3830	3898/3898/3898/3899/3900/3897/3899	70/70/70/70/70/70/70	3/3/3/3/3/3/3			H
*trnQ*	3898/3898/3898/3899/3900/3897/3899	3968/3968/3968/3969/3970/3967/3969	71/71/71/71/71/71/71	-1/-1/-1/-1/-1/-1/-1			L
*trnM*	3968/3968/3968/3969/3970/3967/3969	4036/4036/4036/4037/4038/4035/4037	69/69/69/69/69/69/69	-1/-1/-1/-1/-1/-1/-1			H
*nad2*	4037/4037/4037/4038/4039/4036/4038	5082/5082/5082/5083/5084/5081/5083	1046/1046/1046/1046/1046/1046/1046	0/0/0/0/0/0/0	ATG/ATG/ATG/ATG/ATG/ATG/ATG	TA/TA/TA/TA/TA/TA/TA	H
*trnW*	5083/5083/5083/5084/5085/5082/5084	5154/5154/5154/5155/5156/5153/5155	72/72/72/72/72/72/72	0/0/0/0/0/0/0			H
*trnA*	5156/5156/5156/5157/5158/5155/5157	5224/5224/5224/5225/5226/5223/5225	69/69/69/69/69/69/69	1/1/1/1/1/1/1			L
*trnN*	5226/5226/5226/5227/5228/5225/5227	5298/5298/5298/5299/5300/5297/5299	73/73/73/73/73/73/73	1/1/1/1/1/1/1			L
*trnC*	5334/5334/5334/5335/5336/5333/5335	5399/5399/5399/5400/5400/5398/5400	66/66/66/66/65/66/66	35/35/35/35/35/35/35			L
*trnY*	5400/5400/5400/5401/5401/5399/5400	5469/5469/5469/5470/5470/5468/5469	70/70/70/70/70/70/70	0/0/0/0/0/0/-1			L
*cox1*	5471/5471/5471/5472/5472/5470/5471	7066/7066/7066/7067/7067/7020/7066	1596/1596/1596/1596/1596/1551/1596	1/1/1/1/1/1/1	GTG/GTG/GTG/GTG/GTG/GTG/GTG	TAA/TAA/TAA/TAA/TAA/TAA/TAA	H
*trnS2*	7067/7067/7067/7068/7068/7045/7067	7137/7137/7137/7138/7138/7115/7137	71/71/71/71/71/71/71	0/0/0/0/0/24/0			L
*trnD*	7141/7141/7141/7142/7142/7119/7141	7213/7213/7213/7214/7214/7191/7213	73/73/73/73/73/73/73	3/3/3/3/3/3/3			H
*cox2*	7219/7219/7219/7220/7220/7197/7219	7909/7909/7909/7910/7910/7887/7909	691/691/691/691/691/691/691	5/5/5/5/5/5/5	ATG/ATG/ATG/ATG/ATG/ATG/ATG	T/T/T/T/T/T/T	H
*trnK*	7910/7910/7910/7911/7911/7888/7910	7983/7983/7983/7984/7984/7961/7983	74/74/74/74/74/74/74	0/0/0/0/0/0/0			H
*atp8*	7985/7985/7985/7986/7986/7963/7985	8152/8152/8152/8153/8153/8130/8152	168/168/168/168/168/168/168	1/1/1/1/1/1/1	ATG/ATG/ATG/ATG/ATG/ATG/ATG	TAA/TAA/TAA/TAA/TAA/TAA/TAA	H
*atp6*	8143/8143/8143/8144/8144/8121/8143	8826/8826/8826/8827/8827/8804/8826	684/684/684/684/684/684/684	-10/-10/-10/-10/-10/-10/-10	ATG/ATG/ATG/ATG/ATG/ATG/ATG	TAA/TAA/TAA/TAA/TAA/TAA/TAA	H
*cox3*	8826/8826/8826/8827/8827/8804/8826	9609/9609/9609/9610/9610/9587/9609	784/784/784/784/784/784/784	-1/-1/-1/-1/-1/-1/-1	ATG/ATG/ATG/ATG/ATG/ATG/ATG	T/T/T/T/T/T/T	H
*trnG*	9610/9610/9610/9611/9611/9588/9610	9681/9681/9681/9682/9682/9659/9681	72/72/72/72/72/72/72	0/0/0/0/0/0/0			H
*nad3*	9682/9682/9682/9683/9683/9660/9682	10030/10030/10030/10031/10031/10008/10030	349/349/349/349/349/349/349	0/0/0/0/0/0/0	ATG/ATG/ATG/ATG/ATG/ATG/ATG	T/T/T/T/T/T/T	H
*trnR*	10031/10031/10031/10032/10032/10009/10031	10099/10099/10099/10100/10100/10077/10099	69/69/69/69/69/69/69	0/0/0/0/0/0/0			H
*nad4l*	10100/10100/10100/10101/10101/10078/10100	10396/10396/10396/10397/10397/10374/10396	297/297/297/297/297/297/297	0/0/0/0/0/0/0	ATG/ATG/ATG/ATG/ATG/ATG/ATG	TAA/TAA/TAA/TAA/TAA/TAA/TAA	H
*nad4*	10390/10390/10390/10391/10391/10368/10390	11770/11770/11770/11771/11771/11748/11770	1381/1381/1381/1381/1381/1381/1381	-7/-7/-7/-7/-7/-7/-7	ATG/ATG/ATG/ATG/ATG/ATG/ATG	T/T/T/T/T/T/T	H
*trnH*	11771/11771/11771/11772/11772/11749/11771	11839/11839/11839/11840/11840/11817/11839	69/69/69/69/69/69/69	0/0/0/0/0/0/0			H
*trnS1*	11840/11840/11840/11841/11841/11818/11840	11906/11905/11906/11907/11907/11884/11906	67/66/67/67/67/67/67	0/0/0/0/0/0/0			H
*trnL1*	11911/11910/11911/11912/11912/11889/11911	11983/11982/11983/11984/11984/11961/11983	73/73/73/73/73/73/73	4/4/4/4/4/4/4			H
*nad5*	11984/11983/11984/11985/11985/11962/11984	13822/13821/13822/13823/13823/13800/13822	1839/1839/1839/1839/1839/1839/1839	0/0/0/0/0/0/0	ATG/ATG/ATG/ATG/ATG/ATG/ATG	TAA/TAA/TAA/TAA/TAA/TAA/TAA	H
*nad6*	13819/13818/13819/13820/13820/13797/13819	14340/14339/14340/14341/14341/14318/14340	522/522/522/522/522/522/522	-4/-4/-4/-4/-4/-4/-4	ATG/ATG/ATG/ATG/ATG/ATG/ATG	TAA/TAA/TAA/TAA/TAA/TAA/TAA	L
*trnE*	14341/14340/14341/14342/14342/14319/14341	14409/14408/14409/14410/14410/14387/14409	69/69/69/69/69/69/69	0/0/0/0/0/0/0			L
*cytb*	14414/14413/14414/14415/14415/14392/14414	15554/15553/15554/15555/15555/15532/15554	1141/1141/1141/1141/1141/1141/1141	4/4/4/4/4/4/4	ATG/ATG/ATG/ATG/ATG/ATG/ATG	T/T/T/T/T/T/T	H
*trnT*	15555/15554/15555/15556/15556/15533/15555	15626/15625/15626/15627/15627/15604/15626	72/72/72/72/72/72/72	0/0/0/0/0/0/0			H
*trnP*	15627/15626/15627/15628/15628/15605/15627	15696/15695/15696/15697/15696/15673/15696	70/70/70/70/69/69/70	0/0/0/0/0/0/0			L
CR	15697/15696/15697/15698/15697/15674/15697	16587/16582/16585/16586/16586/16562/16580	891/887/889/889/890/889/884	0/0/0/0/0/0/0			

### ﻿Nucleotide composition

The nucleotide composition of the seven newly sequenced *Lamprologus* and *Neolamprologus* mitogenomes were biased toward A and T (Table 2). The AT-skews exhibited inconspicuously positive values, while all GC-skews were markedly negative. The analysis revealed a clear preference for the utilization of C, along with a minor inclination towards A, across the entire genome (Table 2).

**Table 2.** Base compositions of the complete genomes, PCGs, rRNAs, tRNAs, and CRs of the seven newly sequenced mitogenomes.

Species	Whole genome		AT - skew	GC - skew	PCGs		tRNAs		rRNAs		CR	
	Size	AT			Size	AT	Size	AT	Size	AT	Size	AT
	(bp)	(%)			(bp)	(%)	(bp)	(%)	(bp)	(%)	(bp)	(%)
*Lamprologuskungweensis*	16,587	54.1	0.002	-0.300	11,466	53.5	1,554	54.7	2,613	54.1	891	62.9
*Lamprologusmeleagris*	16,582	55.1	0.002	-0.300	11,466	54.7	1,553	55.8	2,613	54.1	887	63.6
*Lamprologusornatipinnis*	16,585	53.9	0.006	-0.304	11,466	53.2	1,554	55.4	2,613	53.5	889	62.7
*Neolamprologusbrevis*	16,586	53.6	0.011	-0.311	11,466	53.0	1,554	54.9	2,614	53.0	889	63.8
*Neolamprologuscaudopunctatus*	16,586	53.9	0.002	-0.299	11,466	53.2	1,552	55.4	2,613	53.2	890	63.0
*Neolamprologusleleupi*	16,562	53.7	0.017	-0.318	11,421	53.0	1,553	54.7	2,612	53.0	889	62.5
*Neolamprologussimilis*	16,580	54.1	0.010	-0.311	11,466	53.5	1,554	54.9	2,598	53.9	884	63.0

**Figure 2.**
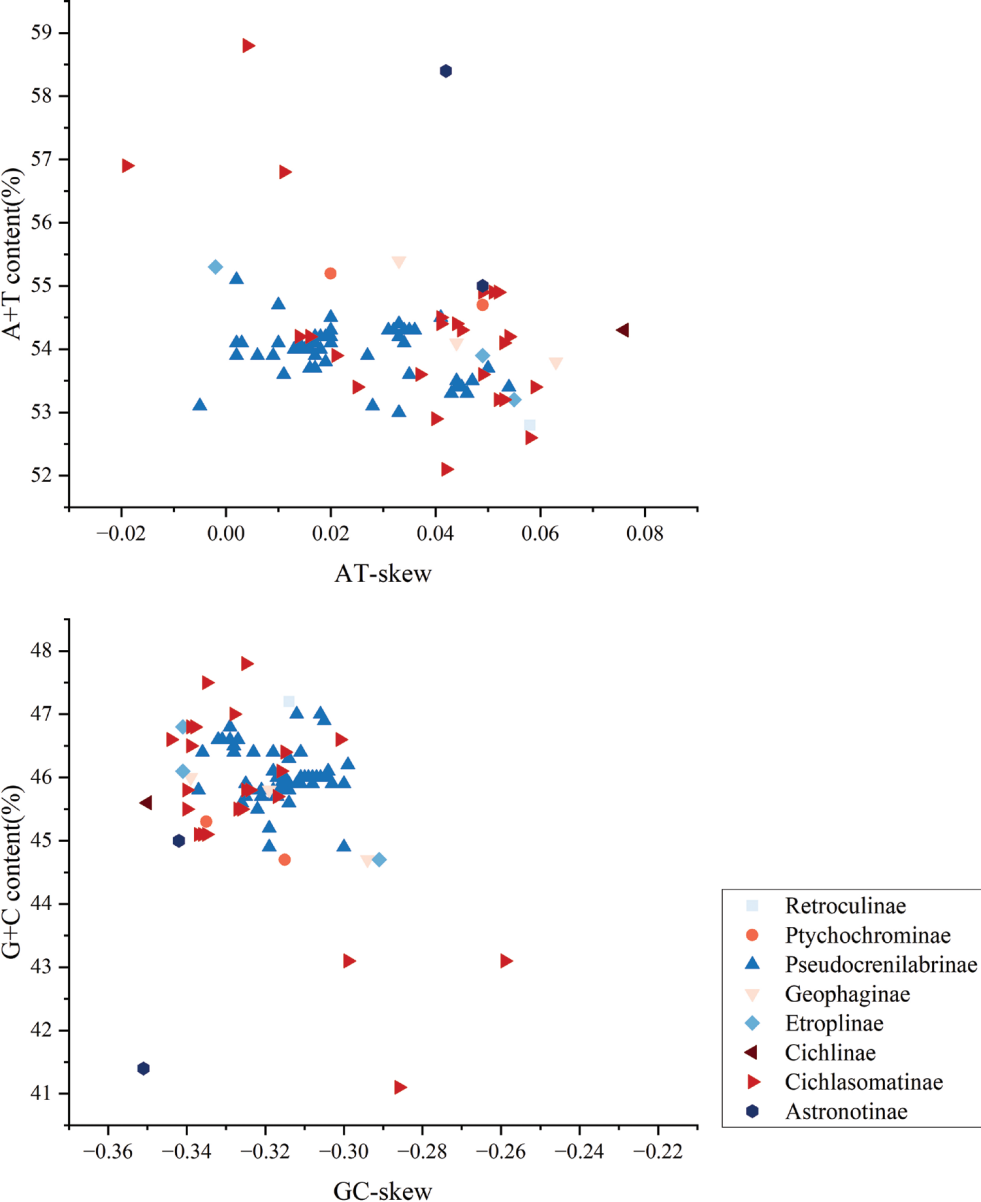
A + T content vs AT-skew and G + C content vs GC-skew in the 103 mitogenomes of family Cichlidae. Values are calculated on H-strands for full-length mitogenomes.

To determine the nucleotide composition of Cichlidae, the A + T content, AT-skew, G + C content, and GC-skew of 103 complete mitogenomes (including 8 subfamilies Astronotinae, Cichlasomatinae, Cichlinae, Etroplinae, Geophaginae, Pseudocrenilabrinae, Ptychochrominae, and Retroculinae of the family Cichlidae) were calculated. The H-strand in the mitogenomes of 103 cichlid species showed a similar preference for A and T nucleotides. The 103 Cichlidae mitogenomes had a comparable nucleotide composition, A + T content (52.1 ~ 58.8%) were higher than the G + C content (41.1 ~ 47.8%) (Fig. 2). The GC-skew were negative (–0.351 ~ –0.221), indicating a higher occurrence of C than G except for *Andinoacararivulatus* (–0.019), *Pelvicachromispulcher* (–0.005), and *Etropluscanarensis* (–0.002). The AT-skew were inconspicuously positive (0.002 ~ 0.076), indicating a small difference in the content of A and T in the mitogenomes. This phenomenon is also observed in other published Teleostei genomes (Liu et al. 2020; Ruan et al. 2020; Xu et al. 2021a, 2021b; Wang et al. 2023). The A nucleotide composition is commonly used to indicate gene direction and replication orientation during transcription and replication (Wei et al. 2010a, 2010b).

### ﻿Protein-coding genes

In the seven newly sequenced mitogenomes, PCG *nad6* was on the L-strand, while other PCGs were on the H-strand. The average A + T content of the PCGs ranged from 53.0% (*N.leleupi* and *N.brevis*) to 54.7% (*L.meleagris*). Six of them had the same 13 PCGs length of 11,466 bp, while the remaining species, *N.leleupi*, had a slightly shorter length of 11,421bp. The reason for this difference was that the *cox1* gene in *N.leleupi* had a mutation causing a premature stop codon compared to other species, resulting in a reduction of 45 base pairs in length (Tables 1, 2).

**Figure 3.**
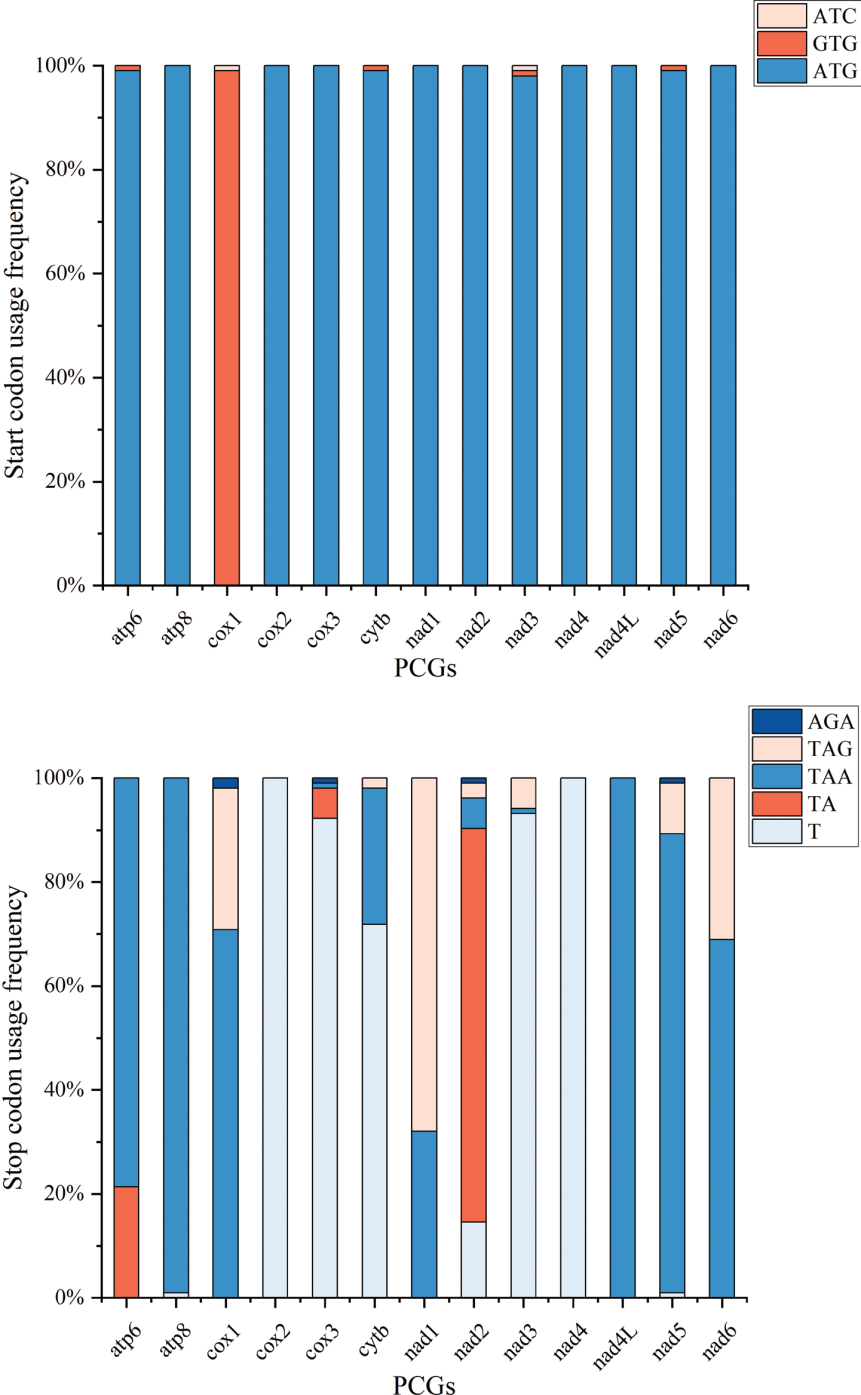
Start codon and stop codon usage for the mitochondrial genome protein-coding genes of 103 cichlid species.

Most of the PCGs in the seven newly sequenced mitogenomes began with the start codon ATG, except for *cox1*, which started with GTG. Most PCGs terminated with the codon TAA or incomplete codon (TA− / T−−), with the exception of *nad1*, which ended with TAG (Table 2). The cichlid species are relatively conservative in their use of start codons, and their preferences are generally consistent with those of the seven newly sequenced species with the only exception of the occurrence of a rare start codon ATC in the *cox1* and *nad3*. All the Cichlids share the stop codons with TAA, TAG, AGA, and incomplete codons (TA− / T−−) (Fig. 3).

**Figure 4.**
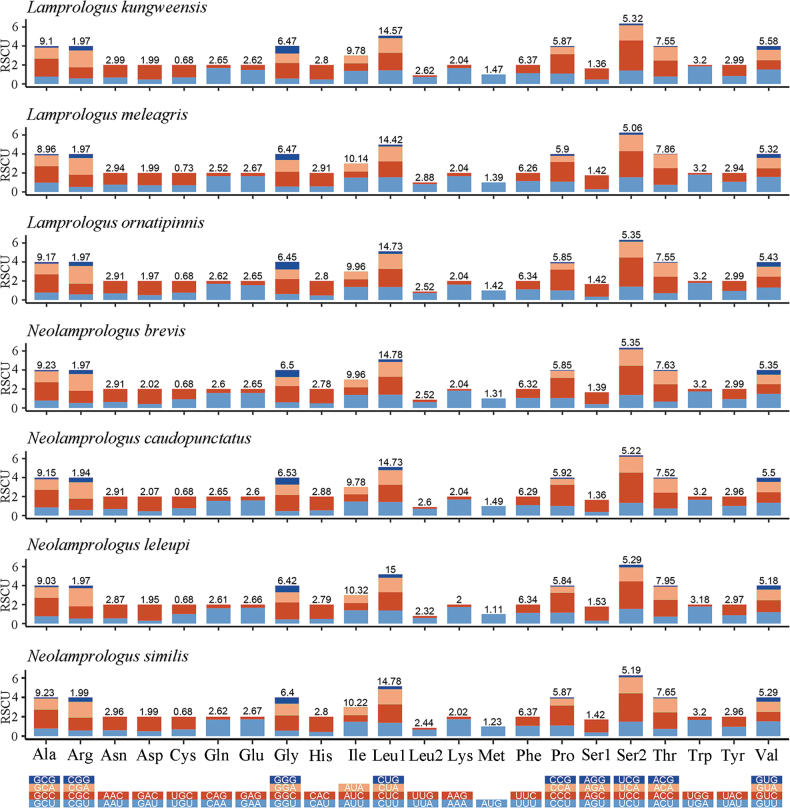
The codon distribution and RSCU of the mitogenomes of the seven newly sequenced mitogenomes.

RSCU was calculated to identify the predominant synonymous codon (Grantham et al. 1980). The comparative analysis based on RSCU of all PCG codons showed that the codon usage patterns of these seven species were similar (Fig. 4). Genes encoding Ile and Leu2 had high frequency, while those encoding Cys, Met, and Ser1 were infrequent.

### ﻿Evolutionary analyses

**Figure 5.**
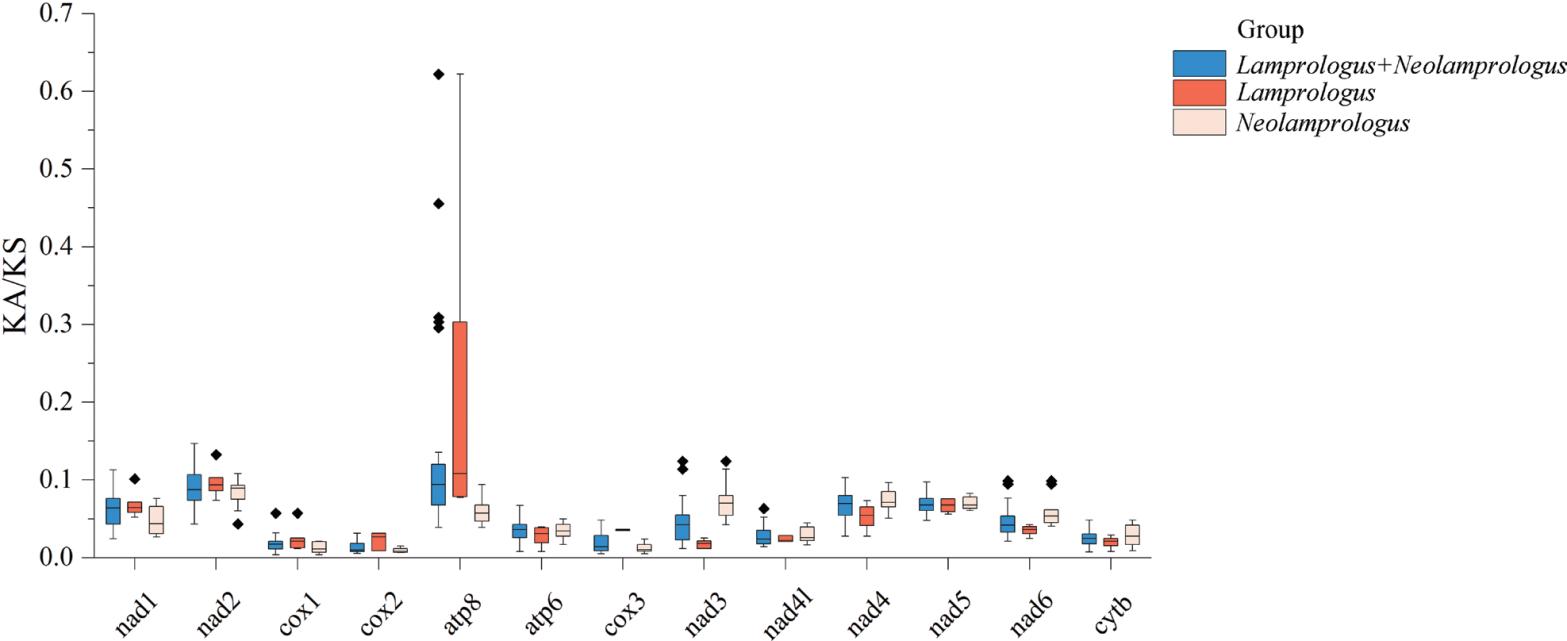
Ka/Ks values for the 13 PCGs. Pale pink box plots, five species of gnus *Neolamprologus*; orange box plots, four species of *Lamprologus*; blue box plots, nine species of *Lamprologus* and *Neolamprologus*. The band inside the box represents the median; upper and lower hinges correspond to the 25^th^ and 75^th^ percentiles; circles, to outliers.

The selection pressure was analyzed by calculating the ratio of Ka/Ks across *Lamprologus* and *Neolamprologus* for each aligned PCG (Fig. 5) (Yang and Nielsen 2002). It was found that *atp8* showed the largest Ka/Ks value among the 13 PCGs, which suggested more amino acid variety in the biomolecule. This suggests that the atp8 gene might have evolved faster than other PCGs due to slight selection pressure (Hassanin et al. 2005). The faster evolution of the atp8 gene could result in greater amino acid diversity, indicating its potential as an effective marker for population classification. The Ka/Ks values for all PCGs were lower than 1, suggesting that purifying selection was likely the main driver of mitochondrial PCG evolution (Hurst et al. 2002).

**Figure 6.**
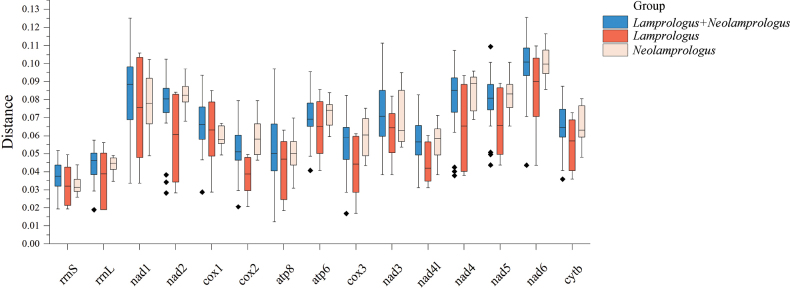
Genetic p-distances for nucleotide sequences for 13 PCGs and 2 rRNAs. Pale pink box plots, five species of *Neolamprologus*; orange box plots, four species of *Lamprologus*; blue box plots, 9 species of genera *Lamprologus* and *Neolamprologus*.

Besides the Ka/Ks analysis, an assessment of the degree of divergence in *Lamprologus* and *Neolamprologus* was conducted by analyzing the overall p-distance between nucleotides of 13 PCGs + two rRNA genes (Fig. 6). The results of p-distance indicated that the fastest-evolving gene was *nad6*, which was inconsistent with the results of Ka/Ks value. However, the difference in this gene might be not comparable with the selection since this force is acting in a contemporary period.

### ﻿Ribosomal RNA genes, transfer RNA genes, and control regions

The size of the *rrnS* genes were between 943 bp (*L.meleagris*, *N.brevis*, and *N.caudopunctatus*) and 946 bp (*N.similis*), while the size of the *rrnL* genes in seven species ranged between 1,652 bp (*N.similis*) to 1,671 bp (*N.brevis*) (Table 1). The two rRNA genes located between *trnF* and *trnL2*, with *trnV* separating them. The A + T content of rRNAs ranged from 53.0% ~ 54.1% (Table 2).

The sizes of the tRNA genes ranged from 66 bp (*trnY* of *N.caudopunctatus*) to 74 bp (*trnK*). The combined length of the 22 tRNA genes varied between 1,552 bp (*N.caudopunctatus*) and 1,554 bp (*L.kungweensis*, *L.ornatipinnis*, and *N.similis*). The A + T contents of tRNA genes ranged from 54.7% to 55.8% among the seven species analyzed in this study (Table 2).

As with other fish mitogenomes, the CRs were discovered to exist between *trnF* and *trnP* in all seven species. The sizes of the CRs ranged from 884 bp (*N.similis*) to 891 bp (*L.kungweensis*). The A + T contents of PCGs, tRNAs, and rRNAs sequences were found to be similar to that of the entire mitogenomes, whereas CR sequences had a higher A + T content (62.5% ~ 63.8%) (Table 2).

### ﻿Phylogenetic analysis

**Figure 7.**
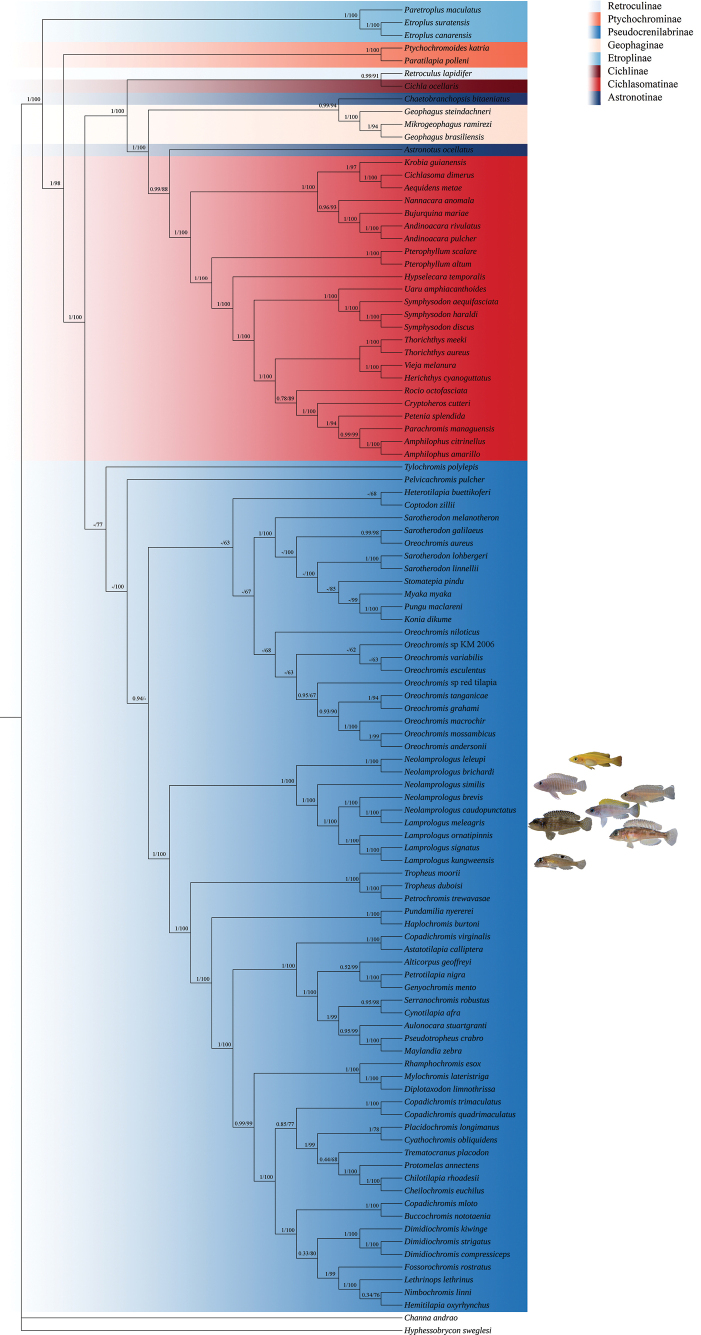
13 PCGs-based phylogenetic tree of 103 cichlid species and two outgroups. Numbers at nodes represent the posterior probability and bootstrap values for BI and ML analysis, respectively. “-” indicates this clade not supported by BI or ML analysis.

To elucidate the phylogenetic inter-relationships within the family Cichlidae and genera *Lamprologus* and *Neolamprologus*, concatenated nucleotide sequences of 13 PCGs + two rRNAs from 103 cichlid species were obtained. Additionally, *Channaandrao*, and *Hyphessobryconsweglesi* from two other families were used as outgroups. It was found that BI and ML analysis generated the same topology structure on most nodes (Fig. 7).

Specifically, the seven complete mitogenomes covered two genera in this study have good clustering in phylogenetic trees, and within the family Cichlidae, the subfamily Etroplinae and Ptychochrominae were monophyletic across analyses. They diverged with species in other subfamilies early in the evolutionary history of cichlid fishes. This result was similar to a previous molecular phylogenetic study (Astudillo-Clavijo et al. 2022). Thirty-one species from subfamily Astronotinae, Cichlasomatinae, Cichlinae, Geophaginae, and Retroculinae were clustered into one branch, indicating these five subfamilies were closely related. Moreover, 67 Pseudocrenilabrinae species also formed a monophyletic clade. Pseudocrenilabrinae tribes and their interrelationships were for the most part well supported as reported by Astudillo-Clavijo et al. (2022). Due to the addition of seven newly sequenced mitogenomes, three pairs of sisters (*N.brichardi* + *N.leleupi*, *N.caudopunctatus* + *L.meleagris*, and *L.signatus* + *L.kungweensis*) were newly identified, as shown in Fig. 7. *Lamprologusmeleagris* did not cluster with *Lamprologus* species, but with species from the genus *Neolamprologus*. Previous studies have identified such taxonomic issues in the genera *Lamprologus* and *Neolamprologus* (Schelly et al. 2006; Sturmbauer et al. 2010). Sturmbauer et al. (2010) think a viable way might be to re-assign the genus name *Lamprologus* to most *Neolamprologus* species. Our results also support this scenario. However, the species from the genera *Lamprologus* and *Neolamprologus* used in this study were limited, making it impossible to perform a more detailed analysis. Therefore, to better understand the relationships between members of these two genera, it will be beneficial to include more species in future studies.

In conclusion, our study increased the database of mitogenome in Cichlidae, and showed that mitogenome sequences are efficient molecular markers for studying the phylogenetic relationships within Cichlidae. However, there is a lack of analyses in nuclear genes. In the future study, we will further improve these deficiencies.

## ﻿Additional information

### Author ORCIDs

Jiachen Wang https://orcid.org/0000-0002-0437-7687

Jingzhe Tai https://orcid.org/0009-0001-4507-9280

Wenwen Zhang https://orcid.org/0000-0003-0142-9469

Ke He https://orcid.org/0000-0001-6446-9439

Hong Lan https://orcid.org/0009-0004-2188-3604

Hongyi Liu https://orcid.org/0000-0003-2081-5779

### Data availability

All of the data that support the findings of this study are available in the main text or Supplementary Information.

## Supplementary materials

Supplementary material 1 Summary of the mitochondrial genomes used for phylogenetic analysis Data type xlsx https://binary.pensoft.net/file/937390This dataset is made available under the Open Database License (http://opendatacommons.org/licenses/odbl/1.0/). The Open Database License (ODbL) is a license agreement intended to allow users to freely share, modify, and use this Dataset while maintaining this same freedom for others, provided that the original source and author(s) are credited.Jiachen Wang, Jingzhe Tai, Wenwen Zhang, Ke He, Hong Lan, Hongyi Liu
